# How does social capital shape trajectories of adolescent self‐rated health in the United Kingdom?

**DOI:** 10.1111/jora.70238

**Published:** 2026-07-21

**Authors:** Bill Calvey, James Laurence

**Affiliations:** ^1^ Social Research Institute University College London London UK

**Keywords:** adolescents, autoregression, psychosocial health, self‐rated health, social capital, structural equation modeling, United Kingdom

## Abstract

Adolescent self‐rated health (SRH) is a robust predictor of long‐term health, but less is known about how local psychosocial resources influence its development in adolescence. We explored whether social capital, captured as both household‐level (i.e., parents' social embeddedness in their neighborhood) and community‐level (i.e., average stocks of social capital in the area), influences trajectories of SRH in early and middle adolescence in the United Kingdom. Using data from the UK Household Longitudinal Study, we tracked a sample of 2413 adolescents, aged 10–15 years at baseline, over a 5‐year follow‐up period. Latent Variable Autoregressive Latent Trajectory (LV‐ALT) models, within a structural equation modeling framework, captured both the stability and changeability of SRH over time, assessing the influence of baseline levels of social capital, adjusting for key sociodemographic and psychosocial characteristics. SRH showed high stability at the population level over 5 years. Higher household “cognitive” social capital scores at baseline were associated with better initial SRH (*β* = .09, *p* < .01) and higher household “network” social capital predicted positive changes in SRH over time (*β* = .02, *p* < .05). Community‐level social capital was not associated with either baseline or changing SRH. Girls reported lower baseline SRH (*β* = −.08, *p* < .001) and experienced more pronounced declines over time than boys (*β* = −.05, *p* < .001). Overall, household‐level social capital, specifically parental embeddedness within local neighborhoods, plays distinctive roles in supporting early and middle adolescent SRH, highlighting the importance of local family and neighborhood connections for healthier trajectories. Investing in these forms of social capital may promote healthier adolescent development.

## INTRODUCTION

Self‐rated health (SRH) is a widely implemented, single‐item measure that requires individuals to appraise their overall health status, with responses typically ranging from “poor” to “excellent.” SRH integrates biological, psychological and social health information into a powerful, unified assessment (Cullati et al., 2020; Picard et al., 2013), which has become fundamental for epidemiologic and population health surveys. In adolescence, poor SRH has been linked to an increased risk of premature mortality and future morbidities in adulthood (Hetlevik et al., 2020; Larsson et al., 2002; Vie et al., 2019), while also being associated with depressive symptomatology (Okada et al., 2022) and unfavorable health behaviors such as physical inactivity and smoking (Lachytova et al., 2017; Zhang et al., 2020).

Although SRH is a robust prognostic tool, SRH remains under‐researched in adolescent populations (Boardman, 2006), despite adolescence being a critical window for the formation of health perceptions (Riley, 2004). Early‐to‐mid adolescence is characterized by neurobiological maturation and major social role transitions (Sawyer et al., 2018), during which young people move away from parental frameworks and increasingly evaluate their health in relation to peer norms and broader social contexts (Sawyer et al., 2018). At the same time, key health behaviors, such as physical activity, diet, substance use, and the management of chronic conditions, begin to consolidate and are shaped by these emerging social environments (Borraccino et al., 2019; Mackenbach et al., 2008; Piko, 2007). Within this developmental context, adolescents must learn how to integrate emerging bodily sensations, affective states and social feedback into a coherent self‐evaluation of health (Joffer et al., 2016). This change suggests that SRH in adolescence may be less crystallized and more sensitive to psychosocial influences than in adulthood. Given that many adult diseases trace their origins back to biological and behavioral processes initiated in adolescence (Paus et al., 2008; Sawyer et al., 2012; Tohi et al., 2022), understanding the psychosocial context in which SRH develops is vital. Yet, an insufficient breadth of research has examined how modifiable psychosocial determinants longitudinally shape adolescent SRH (Viner et al., 2012), a gap the present study seeks to address.

Prior work has outlined several conceptual frameworks that elucidate how SRH evolves over the life course. Firstly, SRH is often conceptualized as an *enduring*, trait‐like construct (Boardman, 2006), where an individual develops a relatively stable, long‐term narrative shaped by past and present experiences as well as expectations for future health (Bailis et al., 2003; Boardman, 2006; Jylhä, 2009). From this perspective, a general sense of health may emerge early in life and persist over time (Bailis et al., 2003; Bollen & Gutin, 2021). Secondly, SRH may function as a *spontaneous* self‐assessment of current health status, influenced by proximal factors present at the time of reporting. For example, adolescents may base their SRH responses on immediate somatic cues, such as pain levels, minor ailments or recent changes in objective health status (Bailis et al., 2003; Perruccio et al., 2010).

Thirdly, SRH is being increasingly recognized as a *lagged* (or autoregressive) construct, whereby current SRH scores are influenced by prior evaluations (Jylhä, 2009; Kosloski et al., 2005; Liu et al., 2023). This perspective differs fundamentally from both the *enduring* view (which attributes stability to long‐term trait continuity) and the *spontaneous* view (where ratings reflect immediate, real‐time health experiences). Instead, the *lagged* perspective suggests that stability in SRH is driven by short‐term anchoring, where individuals actively use recent self‐ratings of health as a cognitive reference point to form their current response (Bollen & Gutin, 2021), highlighting SRH as an autoregressive construct. Fourthly, a *life course* perspective emphasizes that SRH is shaped by age, period and cohort‐specific influences, recognizing that health perceptions evolve in response to changing developmental and social contexts (Elder et al., 2003; Shanahan, 2000). As an individual progresses through the lifespan, major life events (such as completing education, experiencing parenthood, entering retirement or navigating crises like pandemics) can profoundly shape and complicate SRH responses. SRH evaluations are therefore not formed in a vacuum, but are bounded by an individual's unique accumulation of lived experiences.

Although *enduring, spontaneous, lagged* and *life course* explanations of SRH offer a useful heuristic, their applicability to adolescence is less clear, with most studies assessing change throughout adulthood. Some empirical evidence suggests that SRH has a dual nature in adolescence, where it exhibits both *enduring* and *spontaneous* qualities (Boardman, 2006). For example, changes in well‐being (e.g., the onset of, or transition away from, a particular illness) do not always result in corresponding changes in SRH (Leinonen et al., 1998), implying that SRH may be more of an *enduring* self‐concept, that is only partially influenced by these *spontaneous* qualities. Another investigation found that models incorporating both *lagged* and *enduring* components best described change in SRH from adolescence to midlife (Bollen & Gutin, 2021). However, given that health self‐concepts are still forming and are relatively malleable in adolescence (Riley, 2004), it is plausible that previous studies may have underplayed the influence that *spontaneous* assessments of SRH have during adolescence, as adolescents respond to their rapidly changing physical, emotional and social environments. At the same time, the emergence of *lagged* effects may not fully develop until late adolescence, when health self‐concepts are more developed. Finally, Bollen and Gutin (2021) suggested that all four perspectives may not be mutually exclusive, with a combination of all approaches potentially informing the evolution of SRH from adolescence into adulthood.

Although SRH is traditionally portrayed as a relatively stable construct from adolescence into adulthood (Breidablik et al., 2008; Fosse & Haas, 2009; Jylhä, 2009; Vie et al., 2014), emerging longitudinal research indicates that it retains a considerable degree of changeability. Recent studies have challenged this assumption of uniform stability, suggesting that SRH can follow a curvilinear trajectory from mid‐adolescence into early adulthood (Sokol et al., 2017). From early adolescence, different trajectories in SRH begin to emerge, with boys generally showing more stable patterns over time, while girls tend to experience steeper declines that persist into young adulthood (Cosma et al., 2021; Haugland et al., 2001). (Cosma et al., 2021; Haugland et al., 2001). Moreover, Bauldry et al. (2012) demonstrated that over a 14‐year period, males, White individuals, those who lived with two parents, and those whose parents had higher educational attainment consistently reported higher SRH than others.

Ultimately, the changeable aspects of adolescent SRH are often attributed to a complex interplay of sociodemographic (e.g., gender, age, family structure), lifestyle (e.g., physical activity, smoking, diet) and psychosocial and contextual factors (e.g., family support, neighborhood social capital, close friendships) (Breidablik et al., 2008; Burdette et al., 2017; Craig et al., 2018; Meland et al., 2020). Over the past three decades, theorists have argued that enhancing (adolescent) health requires focusing not just on an individual's traditional risk or protective factors, but also on the social environments in which individuals are embedded and that shape their chances of being healthy – commonly referred to as the social determinants of health (Sawyer et al., 2012). Studies within this framework examine how differences between individuals relate to differences between populations and how social gradients and cultural factors affect health outcomes (Marmot et al., 2008).

Among these broader psychosocial determinants of health, social capital may play a crucial role in shaping the trajectories of adolescent SRH. Social capital is broadly defined as the networks, norms and trust that facilitate cooperation for mutual benefit (Kawachi et al., 1997; Putnam, 2000). It can be measured in various settings such as the household, neighborhood or school contexts (Harpham et al., 2002) or measured at both the individual‐ (e.g., number of close friends or perceived belonging within a neighborhood) and the community‐level (e.g., household‐ or community‐level social connectivity). A growing body of evidence suggests that social capital benefits health and quality of life through mechanisms such as buffering stress, providing access to social support, encouraging healthy lifestyles and fostering a sense of belonging and security (De Silva et al., 2006; Kang et al., 2023; Laurence & Calvey, 2025; Nieminen et al., 2013; Poortinga, 2006). Health behaviors themselves are also shaped and constrained by social and community contexts, with individuals' relationships to social networks exerting important influences on health (Baum, 2000; Campbell et al., 1999).

Social capital may further promote collective efficacy within communities, whereby adults monitor, support and transmit shared norms to young people, shaping adolescents' health behaviors, stress exposure and subjective evaluations of health (Sampson et al., 1997; Viner et al., 2012). Empirical evidence from Eastern European, Scandinavian and South American populations supports this perspective, suggesting positive associations between adolescent SRH and various dimensions of social capital (i.e., neighborhood security, reciprocity, social participation, interpersonal trust) (Borges et al., 2010; Novak et al., 2017; Novak et al., 2018; Winding et al., 2025). Conceptually, young people with greater access to social networks and resources are more likely to develop as healthy, caring and productive adults (Morgan & Haglund, 2009; Scales, 1999). A previous UK‐based study identified significant cross‐sectional associations between various domains of social capital, adolescent SRH and selected health behaviors (Morgan & Haglund, 2009). However, evidence remains limited regarding how social capital shapes longitudinal change in adolescent SRH.

Furthermore, conflicting results have also been reported in the relationship between social capital and health. For example, Veenstra (2000) found that only social participation was weakly related to health in Canadian adults, with trust and civic participation showing little association. Furthermore, Kennelly et al. (2003) reported minimal effects of social capital on adult population health, comparing findings across 19 countries. Social capital can also facilitate the spread of risky behaviors within groups, such as substance use or unhealthy eating habits (Villalonga‐Olives et al., 2017). Additionally, some research suggests that only adolescents with high offline social capital have optimal mental health compared to those with high online or mixed social capital, indicating that certain types or combinations of social capital may not be protective and could even be associated with more physical or psychological symptoms (Pan et al., 2023).

As a result, the influence that social capital has on the developmental trajectory of SRH in early to mid‐adolescence remains clouded. Furthermore, much of the social capital literature in adolescence has utilized adolescents' self‐reported levels of social capital (perceptions of their neighborhood) (e.g., Novak et al., 2017; Pan et al., 2023), which may conflate subjective health appraisals with subjective evaluations of social capital. However, what may be important for adolescents, especially in the context of SRH, is the social capital which parents/caregivers in adolescents' households have access to, for example, how much their parents/caregivers feel they belong to the area or whether they exchange favors with their neighbors; henceforth, labeled “household‐level social capital.” It may be that households with higher social capital have access to more positive social environments that support physical and psychological well‐being. In particular, parents' or caregivers' access to, and engagement with, social resources in their local social environment may influence adolescents' SRH through pathways such as exposure to social support, stress buffering and access to community resources (Folland, 2008; Laurence & Calvey, 2025).

In addition, beyond the social embeddedness of the adolescent's own household, the wider local area may also provide collective social resources that shape adolescent SRH – this refers to the average stock of social connectivity, associational life, neighborliness, and civic or social participation within a local area; henceforth, labeled “community‐level social capital.” This measure may help reduce endogeneity between subjective evaluations of social capital and subjective appraisals of health, as it is not self‐reported by adolescents, while also distinguishing the social embeddedness of the adolescent's own household from the broader social connectedness of the area in which that household is located. Community‐level social capital may be relevant to adolescent SRH because socially connected communities may be better able to generate informal support, circulate local information, support collective action, improve access to local services and amenities, and foster trust, perceived safety, and mutual re mutual respect (Kawachi & Berkman, 2001). These processes may matter during adolescence, when young people become increasingly oriented toward peers, neighborhoods, and wider social contexts, and when health appraisals are shaped not only by physical health but also by perceived safety, belonging, and access to supportive social environments. By shaping the conditions in which adolescents encounter and respond to stressors, community‐level social capital may also help buffer the psychological consequences of adversity via greater access to informal support and collective efficacy (Zeng & Wu, 2022).

Taken together, adolescent SRH has considerable *stable* and *changeable* components, yet local psychosocial characteristics, like household‐ and community‐level social capital, may have the potential to influence trajectories of SRH over time, particularly during developmentally sensitive periods such as adolescence. As a result, this study addresses these key gaps by modeling household‐ and community‐level social capital as predictors of baseline adolescent SRH and change in adolescent SRH, examining its *enduring*, *spontaneous*, and *autoregressive* properties, in the context of the United Kingdom. By leveraging nationally representative, longitudinal data, this study systematically tracks changes in SRH over time among early‐to‐mid adolescents resident in the United Kingdom, aged 10–15 years old, over a 5‐year follow‐up period. Based on extant literature, we hypothesize that higher social capital (measured at a household‐ and community‐level) will be significantly associated with higher baseline SRH and with positive changes in SRH over time.

## MATERIALS AND METHODS

### Study population

We utilized archived secondary data to respond to our objectives. Data from a multi‐wave, nationally representative, longitudinal household panel survey called the UK Household Longitudinal Study (UKHLS) were used to track changes in adolescent SRH. UKHLS includes longitudinal measurements of multidimensional aspects of health, occupation, education and social life (Buck & McFall, 2012). The study follows its participants of different ages, ethnicities, educational levels and in different locations across the United Kingdom over time, to understand how the UK's population evolves over the life course. The UKHLS Mainstage data initially recruited over 100,000 participants who resided in 40,000 households to participate in the first wave of data collection. The UKHLS sampled participants through a complex, multi‐stage probability sampling design, creating a nationally representative panel of UK households, which was also supplemented by an ethnic minority boost sample. Data were collected via a combination of face‐to‐face, web and telephone interviewing. UKHLS obtained ethical approval from the University of Essex Ethics Committee, with the consent materials made available as part of the online study documentation (https://www.understandingsociety.ac.uk/documentation/mainstage/fieldwork‐documents).

Currently, there are 14 waves of data collection within the Mainstage survey, ranging from Wave 1 (2009–2010) to Wave 14 (2022–2023). We drew upon two specific UKHLS samples, both of whom completed separate surveys: an adult sample (those aged 16+ within a household) and a youth sample (those aged 10–15 years old present in the household). We used a combination of both survey samples to track changes in SRH over time, as adolescents transition to the adult survey upon turning 16. This means that by the final UKHLS wave that we used in our study, the age range of participants would be from 16 years to 21 years old.

We utilized the six most recent UKHLS waves, ranging from Wave 9 (January 2017 to December 2018) to Wave 14 (January 2022 to May 2024), to capture changes in adolescent SRH scores. We chose the most recent UKHLS waves for this analysis in order to (1) reflect contemporary adolescent health perceptions and trajectories, (2) focus on developmental trajectories from mid‐ and late‐adolescence into early adulthood (up to age 21), and (3) limit cohort attrition and measurement heterogeneity that would arise from a substantially longer follow‐up period. From the initial *n* = 2821 adolescents (aged 10–15 years) who participated in UKHLS Wave 9, we excluded participants who only had one timepoint of SRH data between Waves 9 and 14 (*n* = 405). We also excluded participants who did not have sufficient social capital data, which we were unable to trace back to each adolescent, using the unique household identifier numbers (*n* = 5). Ultimately, we arrived at a final sample size of *n* = 2413 early‐to‐mid adolescents (see Supplementary Materials 1), who were aged between 10 and 15 years at our baseline (i.e. wave 9). Those who were excluded from analyses were more likely to be female (*p* < .001, Cramer's *V* = .04), resident in a single‐parent household (*p* < .001, Cramer's *V* = .07), limited a lot by a chronic health condition/disability (*p* < .001, Cramer's *V* = .08) and were older (*p* < .001, Cramer's *V* = .09) than those who were retained for analyses. This study was not pre‐registered.

### Measures

#### Self‐rated health

SRH was captured at each wave using a single item, which asked participants to consider “In general, would you say your health is…,” with responses ranging from “excellent” (coded as 1) to “poor” (coded as 5). We reverse‐coded these SRH scores so that higher scores would indicate better subjective health. Although assessed using a single item and therefore not permitting an estimation of internal consistency, prior work indicates that a single item of SRH demonstrates moderate test–retest reliability over time, as well as satisfactory convergent and predictive validity with other health constructs (Cullati et al., 2020; Zajacova & Dowd, 2011).

#### Social capital

Social capital was captured at two distinct levels: (1) household‐level social capital (reflecting parents or caregivers' personal experiences of social embeddedness within their neighborhood) and (2) community‐level social capital (reflecting broader patterns of interpersonal relationships and civic engagement within local areas).

##### Household‐level social capital

At baseline, parents/caregivers rated their agreement with various statements tapping different dimensions of neighborhood social capital on a 5‐point Likert scale, ranging from 1 (“Strongly agree”) to 5 (“Strongly disagree”). We ran exploratory factor analyses to derive two distinct indices of household‐level, parent‐reported social capital, similar to previous studies (De Silva et al., 2006; Laurence, 2025). An exploratory factor analysis using a promax rotation yielded one “cognitive” component of household‐level social capital (Minimum factor loading: 0.69; Eigenvalue: 1.5; Alpha coefficient: 0.78). This cognitive component comprised three items capturing parents/caregivers' feelings of belonging and identification with their neighborhood: “I feel like I belong to this neighborhood”, “I plan to remain a resident of this neighborhood for a number of years” and “I think of myself as similar to the people that live in this neighbourhood.” The derived cognitive social capital scores were coded so that higher scores indicated higher levels of cognitive social capital.

The exploratory factor analyses also identified a “network” component of household‐level social capital, which focused on parents' and caregivers' perception of quality and function of neighborhood social ties and perceived reciprocal support (Minimum factor loading: 0.54, Eigenvalue: 2.46, Alpha coefficient: 0.83). This network component comprised five items: “The friendships and associations I have with other people in my neighbourhood mean a lot to me,” “If I needed advice about something I could go to someone in my neighbourhood,” “I borrow things and exchange favors with my neighbours,” “I would be willing to work together with others on something to improve my neighbourhood,” and “I regularly stop and talk with people in my neighbourhood.” Similar to the cognitive component, higher scores on the network component indicated higher levels of social capital.

##### Community‐level social capital

Social capital was also measured at the community‐level. Community‐level social capital was assessed using the “Relationships” dimension of the UK Social Fabric Index, a well‐established tool for quantifying community strength and cohesion across local authorities in the United Kingdom (Tanner et al., 2020). The Social Fabric Index comprises four key thematic threads, each reflecting a distinct dimension of community life. The Relationships dimension specifically measures associational life and captures the extent and quality of interpersonal connections within communities. This measure uses a diverse set of objective indicators (e.g., the prevalence of community‐owned pubs and shops, the number of registered local charities, rates of volunteering) and subjective indicators (e.g., residents' expressions of neighborliness) in constructing a score for each UK Local Authority District. By aggregating these metrics, this community‐level metric provides a suitable proxy for the average levels of local social capital, reflecting the density of local networks and civic/social involvement within a given area.

#### Covariates

We adjusted our analyses for a set of sociodemographic, health‐related and psychosocial covariates based on their established association with adolescent SRH. These covariates included: age, gender, ethnicity, highest level of parental education, single‐parent household status, whether participants changed address during the study period, a community‐level economic disadvantage score, the presence of a long‐term limiting health problem or disability, life satisfaction, family support and the number of close friends (Atienza‐González et al., 2020; Breidablik et al., 2008; Breidablik et al., 2009; Meland et al., 2020; Vingilis et al., 2002).

Ethnicity was classified into five categories (White, Black, Asian, Mixed and Other) in line with the UK Office for National Statistics. Parental education was determined by the highest educational attainment of either parent and categorized into four groups: “None/Primary,” “Lower Secondary,” “Upper Secondary,” and “Tertiary.” Single‐parent households were identified via a binary indicator. Participants' residential locations were linked to Lower Super Output Area (LSOA) codes at each UKHLS wave. A change in LSOA code between waves was used as an indicator that adolescents had changed address during the study period, coded as a binary variable (0 = No, 1 = Yes). An index of community‐level economic disadvantage comprised of three area‐level indicators (captured at the Local Authority District level): proportion of residents in social housing (factor loading: 0.60), unemployment rate (0.82), and proportion of female lone‐parent households (0.79). The index demonstrated good internal consistency (Eigenvalue = 1.65, alpha = 0.71).

The presence of a long‐term limiting health problem or disability was self‐reported by participants, with three response options: “None,” “Yes, limited a little,” or “Yes, limited a lot.” Life satisfaction was assessed using a single item, “How do you feel about your life as a whole?”, with responses reverse‐coded to create a scale from 1 to 7 (higher scores indicate greater life satisfaction). Family support was measured using a 3‐point Likert item, asking participants “whether they feel supported by their family members,” with higher scores indicating higher levels of perceived family support. Lastly, participants self‐reported the number of close friends they had, up to a maximum of 10; scores above 10 were capped to address implausibly high outliers.

Although family support and the number of close friends reflect important interpersonal resources available to adolescents (and can be considered components of adolescent social capital), we did not include these variables within our operationalization of social capital. Our study specifically focused on *parent‐reported, household‐level* social capital (tapping into parents/caregivers' social capital embedded in the family environment) and *community‐level* social capital (tapping into contextual forms of social capital from the wider neighborhood). These constructs differ conceptually from adolescents' own interpersonal support networks. As such, we treated adolescent‐reported family support and close friendships as covariates rather than components of the exposure. This approach also reduces potential endogeneity with adolescents' self‐reported health. A list of all measures included in our analyses can be found in Supplementary Materials 2.

### Data analysis

We tested our hypotheses using Latent Variable Autoregressive Latent Trajectory models (LV‐ALT) within a structural equation modeling (SEM) framework. LV‐ALT modeling is a flexible hybrid approach for modeling longitudinal change, integrating features of latent growth curve models and autoregressive panel models (Bollen & Curran, 2004; Selig & Little, 2012). As prior work shows, SRH exhibits both stable and changeable qualities in adolescence, along with a strong autoregressive component. Bollen and Gutin (2021) systematically compared several longitudinal statistical models in their ability to characterize change in SRH from early adolescence into adulthood. This hybrid framework outperformed more traditional latent growth curve, autoregressive panel, and free‐loading models. As such, this hybrid model is particularly suited for modeling stability and change in adolescent SRH and would allow a more accurate test of the effects of social capital.

**FIGURE 1:**
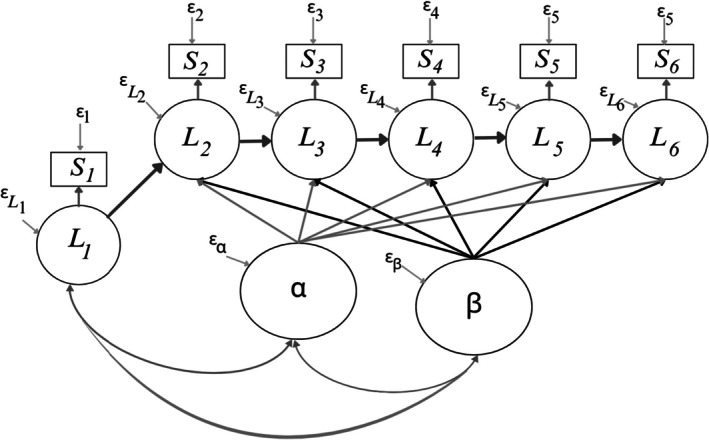
A Linear Latent Variable Autoregressive Latent Trajectory growth model. To simplify the path diagram, we do not visualize the covariates that were regressed onto the slope and intercept factors. *ε* refers to the errors of the underlying self‐rated health variables, as well as the errors for the latent variables. *L*_*t*_ = latent self‐rated health at wave *t*. *S*_*j*_ = measures of self‐rated health at wave *t*; *t* = 1, 2, 3, 4, 5, 6. *α* = latent intercept. *β* = latent linear slope.

SRH was modeled as an observed variable at each wave, with repeated measures decomposed into latent growth components (i.e., intercept and slope). This latent growth structure enables the disaggregation of baseline differences from longitudinal trajectories, acknowledging that respondents' SRH do not change over time in homogeneous ways, but rather, individuals display different starting points, evolving trajectories and rates of change over time that reflect their life and health experiences (Bollen & Brand, 2010; Bollen & Gutin, 2021). LV‐ALT models also incorporate autoregressive pathways into model estimations, whereby SRH at time T is influenced by SRH at time T‐1, recognizing that SRH is largely a *lagged* and *enduring* self‐concept (Boardman, 2006). This kind of latent trajectory model is advantageous over traditional approaches, due to its flexibility regarding missingness, non‐normally distributed repeated measures and accounting for measurement error (Curran et al., 2010). Residual variances were freely estimated at each time point, capturing both measurement error and occasion‐specific variability. Figure 1 is a path diagram which visualizes the structure of our LV‐ALT model.

Analyses were conducted in R Studio using the *lavaan* package (Rosseel, 2012). To determine the growth function that best characterized change in adolescent SRH across six waves, we compared unconditioned linear and non‐linear LV‐ALT models, similar to previous SEM practices (McHugh Power et al., 2023). We used chi‐square tests to assess how closely the hypothesized models fit the UKHLS data, though given the high power of large cohort sample sizes, even minor misspecifications can yield significant results. We therefore also considered Akaike information criterion (AIC) and Bayesian information criterion (BIC) values, with lower scores indicating better fit (Raftery, 1995). We also referred to the comparative fit index (CFI), Tucker‐Lewis index (TLI) and the root mean square error of approximation (RMSEA) when assessing model fit (Bentler, 1990; Tucker & Lewis, 1973). CFI and TLI scores that are closer to 1 indicate better fit, with scores less than 0.9 being inadequate. RMSEA scores closer to 0 indicate better model fit, with values >0.10 indicating poor fit.

Once the appropriate growth function for adolescent SRH was established (i.e. linear or non‐linear), we extended the model to include sociodemographic and psychosocial covariates (including indicators of social capital), which were regressed onto the latent intercept and slope factors. We then ran additional models which tested for interaction effects between gender and various dimensions of social capital, ultimately to determine if the association that social capital has with adolescent SRH varies across boys and girls (see Supplementary Materials 3). Since both cognitive and network social capital scores are expected to be highly correlated, we ran a sensitivity analysis where, instead of cognitive and network social support being parsed out into two separate indices, we included one overall score of household‐level social capital (see Supplementary Materials 4). In all models, missing data were addressed using a full‐information maximum likelihood (FIML), which utilizes all available data to estimate population parameters in the presence of missing data and has been shown to perform equivalently to multiple imputation in handling missingness (Lee & Shi, 2021).

## RESULTS

In a sample of 2413 adolescents resident in the United Kingdom, 51.26% were female, with a mean sample age of 12.49 years (±1.70). Approximately 17.55% of our sample lived in single‐parent households at baseline, with 9.26% being limited a little bit by a long‐term health condition/disability and 1.93% of adolescents being limited a lot by a long‐term health condition/disability. The mean SRH score at baseline was 3.93 (±0.92). Full descriptive statistics are available in Supplementary Materials 5.

We estimated unconditioned linear and non‐linear LV‐ALT models to evaluate which growth function best characterized change in SRH over time, with the caveat that assessing fit in LV‐ALT models can be difficult. Firstly, we ran an unconditioned linear time model, where the values applied to each wave were 1, 2, 3, 4, 5 and 6, corresponding to each UKHLS wave. Model fit in this initial linear model was good (CFI = 0.99, TLI = 0.99, *χ* ^2^ = 12.94, *p* = .23; RMSEA = 0.01). Then, in a separate model, we specified Time as non‐linear, with the values applied to each wave being 0, 1, 4, 9, 16 and 25. Fit was broadly comparable to the linear model across most global fit indices (CFI = 0.99, TLI = 0.99, *χ* ^2^ = 9.33, *p* = .16, RMSEA = 0.02). However, AIC, BIC and RMSEA favored the linear model, indicating that the modest improvement in *χ* ^2^ for the quadratic growth function did not outweigh the increased model complexity (see Table 1). Given this marginal improvement in model fit and the principle of model parsimony, we determined that a linear growth function best described change in SRH over time.

**TABLE 1:** Comparison of fit indicates for LV‐ALT longitudinal models of self‐rated health in adolescents aged 10–15 years: UKHLS, Waves IX‐XIV (2017–2023). Based on the fit indices below, a linear growth function was chosen.

Model	*χ* ^2^	*p*	*df*	AIC	BIC	CFI	TLI	RMSEA
*LV‐ALT Linear Growth*	*12.94*	.*23*	*10*	*24,314*	*24,413*	*0.99*	*0.99*	*0.01*
LV‐ALT Quadratic Growth	9.33	.16	6	24,319	24,440	0.99	0.99	0.02

After establishing that a linear LV‐ALT model provided the more appropriate fit for longitudinal changes in adolescent SRH, we estimated a conditioned model which included key sociodemographic and psychosocial variables (including measures of social capital) measured at baseline, regressed onto the latent intercept and slope factors. This final model demonstrated strong fit (CFI = 0.96, TLI = 0.95, *χ* ^2^ = 140.24, *p* < .001, RMSEA = 0.03).

The mean expected level of SRH scores (intercept) at the beginning of the study was 2.55 (SE = .24, *p* < .001). The mean linear slope was not statistically significant (*β* = −.02, SE = .08, *p* = .67), suggesting that on average, there was no evidence of statistically significant systematic change in SRH over the observed period, after accounting for covariates. Importantly, however, the variance of the slope was statistically significant (*σ* ^2^ = .01, SE = .002, *p* < .001), indicating meaningful between‐individual differences in trajectories of SRH over time. This suggests that, although the average, group level trajectory was stable, adolescents exhibited heterogeneous patterns of change, with some experiencing improvements and others experiencing declines in SRH. Our final model, including covariates and exposure variables, explained 32.9% of the variance in baseline SRH (*R* ^2^ = .329) and 15.1% of the variance in the slope of SRH (*R* ^2^ = .151). There was a negative covariance between the intercept and slope of −.02 (SE = .01, *p* < .001), indicating that adolescents with higher SRH at baseline tended to experience greater declines in SRH over time. The model also revealed increasing autoregressive effects for SRH across successive waves. The influence of prior SRH on subsequent ratings showed no significant association earlier in the study (T2 ~ T1 = .01, *p* > .05), but these autoregressive pathways became statistically significant and grew stronger at each subsequent measurement (T6 ~ T5 = .15, *p* < .001).

**FIGURE 2:**
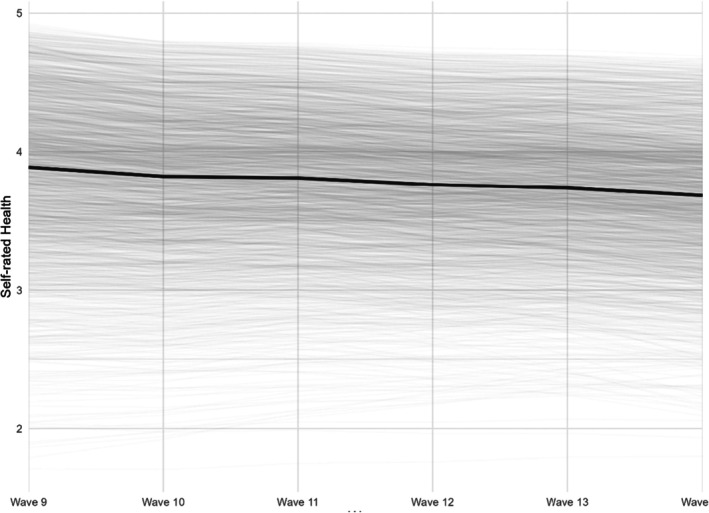
Individual predicted trajectories and mean group change in adolescent self‐rated health over six waves of UKHLS data.

Based on the predicted model values, we plotted the linear trajectory of SRH over time across adolescents (see Figure 2). The bold black line indicates group mean change in SRH over time, while thinner greyscale lines represent various individual changes over time (with overlapping lines darker in color). Evidently, there was minimal wave‐on‐wave change in SRH over time, at a group level. More heterogeneity in the slopes of individuals is observable, but it remained low (variance in slopes across individuals was .03).

Looking into the correlates of the intercept of SRH, younger age (*β* = −.05, *p* < .001), adolescents living in a single‐parent household (*β* = −.10, *p* = .04), being limited a little by a long‐standing disability or illness (*β* = −.32, *p* < .001) and being limited a lot by a long‐standing disability or illness (*β* = −.53, *p* < .001) were all associated with lower SRH at baseline. Additionally, higher levels of family support (*β* = .25, *p* < .001) and life satisfaction (*β* = .18, *p* < .001) were associated with higher baseline levels of SRH. Meanwhile, no statistically significant results were observed for gender, all levels of parental education, economic disadvantage score, and number of close friends.

**FIGURE 3:**
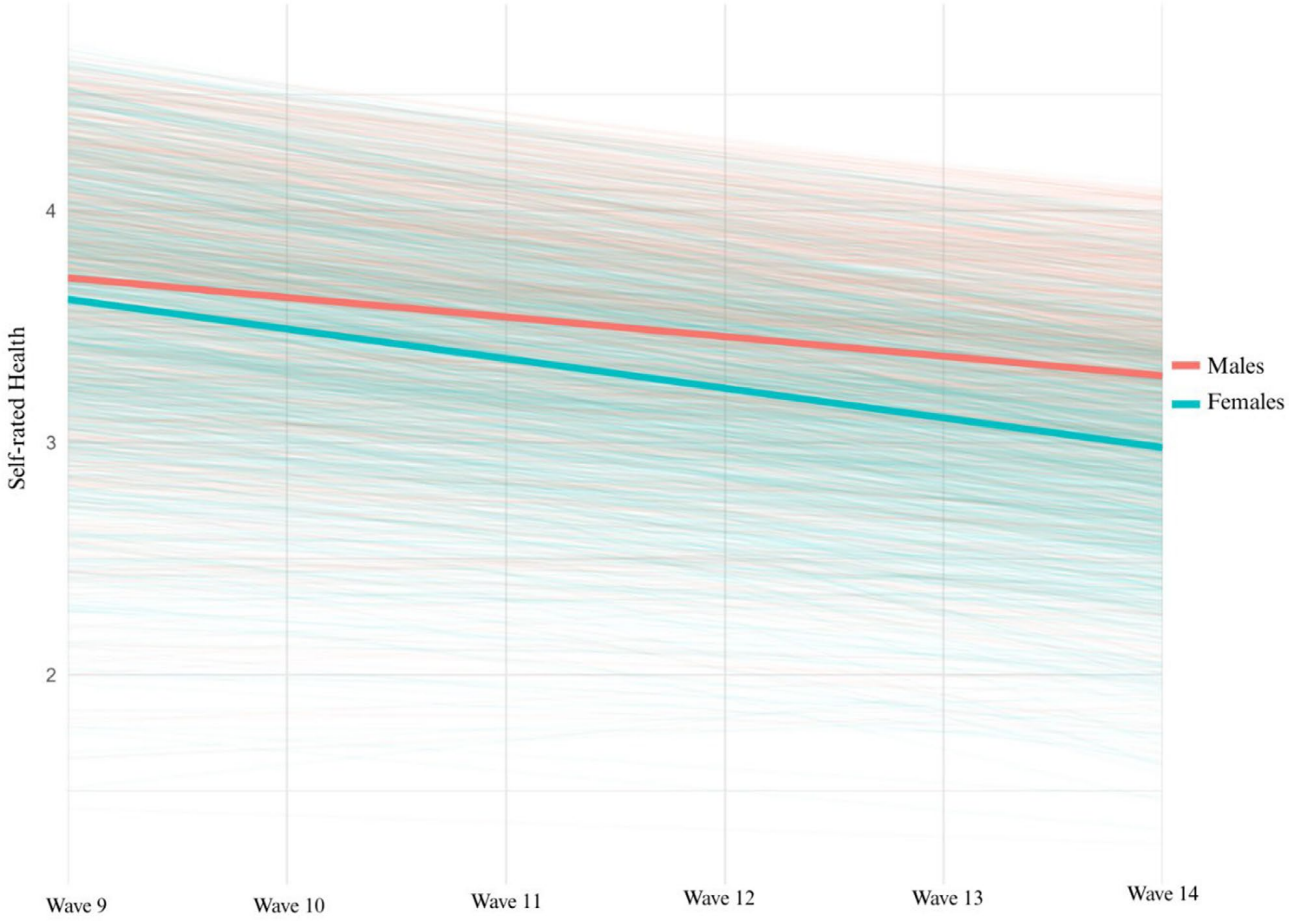
Predicted trajectories of adolescent self‐rated health over six waves of UKHLS data, stratified by gender.

Looking into the correlates of the slope of SRH, older participants showed a less steep decline in SRH than younger participants (*β* = .01, *p* < .001). Girls showed greater declines in SRH than boys over time (*β* = −.05, *p* = .01). Additionally, higher levels of life satisfaction (*β* = −.02, *p* < .001) and family support (*β* = −.03, *p* < .001) were associated with greater declines in SRH over time (see Table 2). We also visualized predicted trajectories across boys and girls in Figure 3. Over the study period, boys experienced an SRH decline of 0.05 units, while girls experienced a more pronounced decline in SRH by about 0.28 units.

**TABLE 2:** A Linear Latent Variable Autoregressive Trajectory model, with the intercept and slope of self‐rated health as outcomes.

	Estimate	Standardized estimate	Std. error	95% CI
*Autoregressive paths*				
T2 ~ T1	.01	.02		
T3 ~ T2	.09	.08		
T4 ~ T3	.11**	.10		
T5 ~ T4	.14**	.12		
T6 ~ T5	.15***	.13		
*Intercept*				
Age	−.05***	−.13	−.12	(−.07/−.03)
Gender (ref = males)	−.08**	−.05	−.03	(−.14/−.02)
Ethnicity (ref = White)				
Black	.05	.04	.08	(−.10/.20)
Asian	−.06	−.05	.05	(−.15/.03)
Mixed	.01	.02	.07	(−.12/.15)
Other	.24	.04	.19	(−.13/.62)
Parental education (ref = none)				
Lower secondary	−.04	−.01	.05	(−.15/.06)
Upper secondary	−.01	−.02	−.06	(−.15/.12)
Tertiary	.05	.05	.04	(−.04/.14)
Economic disadvantage score	−.02	−.06	.03	(−.08/.03)
Single‐parent household (ref = no)	−.10*	−.08	.04	(−.18/−.02)
Change of address	−.02	−.03	.03	(−.19/.06)
Limiting health condition/disability (ref = none)				
Yes, limited a little	−.32***	−.12	.05	(−.42/−.21)
Yes, limited a lot	−.53***	−.13	.11	(−.75/−.31)
Family support	.25***	.16	.04	(.17/.32)
Life satisfaction	.20***	.37	.01	(.17/.23)
Close friends	.01	.04	.01	(−.001/.02)
Household‐level social capital				
Cognitive	.09**	.09	.03	(.03/.15)
Network	−.02	−.03	.03	(−.09/.04)
Community‐level social capital	−.01	−.04	.03	(−.07/.05)
*Slope*				
Age	.01***	.05	.003	(.004/.02)
Gender (ref = males)	−.05***	.05	.01	(−.06/−.03)
Ethnicity (ref = White)				
Black	.04	.05	.03	(−.01/.09)
Asian	.02	.07	.01	(−.01/−.04)
Mixed	.01	−.002	.02	(−.03/−.04)
Other	−.11	−.05	.05	(−.21/−.001)
Parental education (ref = none)				
Lower secondary	.00	−.01	.02	(−.03/.03)
Upper secondary	−.01	.01	.02	(−.02/.05)
Tertiary	−.001	.01	.01	(−.03/.03)
Economic disadvantage score	−.01	−.02	.01	(−.02/.01)
Single‐parent household (ref = no)	.001	.002	.01	(−.02/.03)
Change of address	.01	.04	.02	(−.03/.04)
Limiting health condition/disability (ref = none)				
Yes, limited a little	.01	−.01	.02	(−.02/.04)
Yes, limited a lot	.02	.05	.04	(−.04/.09)
Family support	−.03*	−.09	.01	(−.05/−.01)
Life satisfaction	−.02***	−.18	.01	(−.03/−.01)
Close friends	−.001	−.02	.002	(−.004/.002)
Household‐level social capital				
Cognitive	−.02	−.07	.01	(−.04/.01)
Network	.02*	.16	.01	(.01/.04)
Community‐level social capital	−.01	.01	.01	(−.02/.01)
*Fit indices*				
*χ* ^2^	259.88			
*p*	<.001			
AIC	76,474.62			
BIC	76,804.57			
CFI	.96			
TLI	.93			
RMSEA	.03			

*Note*: T1…T6 refers to the six UKHLS timepoints that were drawn on in this study (UKHLS Wave 9 to 14). Significant at *p < .05, **p < .01, ***p < .001.

### Social capital and adolescent self‐rated health

Household‐level, cognitive social capital scores, as reported by parents and caregivers within the household, were significantly associated with higher baseline levels of SRH (*β* = .09, *p* < .01), while household‐level network social capital or community‐level social capital scores (at baseline) were not significantly associated with the latent intercept factor. Conversely, higher levels of household‐level network social capital at baseline were significantly associated with positive increases in SRH over the study period (*β* = .02, *p* = .04). Meanwhile, neither household‐level cognitive social capital nor community‐level social capital at baseline were significantly associated with change in the slope of SRH over time.

Given the important gender differences in SRH trajectories identified in the prior literature, we also ran additional interaction models to determine whether the cross‐sectional and longitudinal association between social capital and SRH significantly varied across boys and girls. No statistically significant interaction terms were found between gender and cognitive and network social capital and between gender and community‐level social capital (see Supplementary Materials 3). Finally, given that there was a strong, positive correlation between the cognitive and network social capital indices within our sample (*r*(2365) = .77, *p* < .001), we conducted a sensitivity test where the overall household‐level social capital index score was included in the models, instead of the separate cognitive and network indices (see Supplementary Materials 4). We found that higher levels of overall household‐level social capital were associated with higher SRH at baseline (*β* = .05, *p* < .01), but such scores did not significantly predict the latent slope factor, demonstrating the importance of distinguishing between the two dimensions.

## DISCUSSION

SRH has traditionally been regarded as a stable construct during adolescence (Boardman, 2006; Breidablik et al., 2009; Fosse & Haas, 2009). However, the stability of SRH in mid‐adolescence has been questioned in recent years, as some studies identified curvilinear trajectories (Bauldry et al., 2012; Sokol et al., 2017), which vary by sociodemographic characteristics (e.g., age, gender, single‐parent households, etc.) and psychosocial circumstances (e.g., family support, close friendships, and neighborhood social capital resources). Despite these insights, relatively few longitudinal studies have examined how local psychosocial resources such as social capital shape adolescent SRH trajectories over time. The present study aimed to fill these prevailing gaps.

We found that household‐level measures of social capital (i.e., parents' reported levels of “cognitive” and “network” social capital) had different effects on the latent intercept and slope factors of adolescent SRH. Higher cognitive‐based scores were associated with better baseline SRH. Potentially, cognitive social capital may enhance self‐appraisals of health by signaling a secure and stable community context, where families intend on living in the future. Factors such as neighborhood identity, trust and perceived socioeconomic status have been shown to be key correlates of adolescent SRH in previous studies (Goodman et al., 2007; Novak et al., 2015) which might explain why cognitive social capital drives higher baseline SRH levels. This broader household‐level measure of social capital operates above and beyond the immediate support from family and friends, fostering a sense of security and collective belonging which seems to be foundational for adolescents' positive health perceptions (Poortinga, 2006; Putnam, 2000).

On the other hand, the network measure of household‐level social capital was positively associated with the latent SRH slope factor (but not the intercept). Higher baseline levels of this network social capital index predicted positive changes in SRH over the study period. Unlike cognitive aspects, this network index reflects actionable, outward‐facing resources. It is possible that households that have access to advice and collective action facilitate more positive social environments and are more supportive of psychological well‐being. Ultimately, households in the United Kingdom with higher network social capital in their neighborhood may have had access to instrumental, social and emotional resources that cumulatively improve the subjective health of adolescents over time (Novak et al., 2018; Sellström & Bremberg, 2006). These supportive exchanges may not have immediately influenced baseline SRH, but as adolescents encounter challenges or opportunities, strong neighborhood and social ties may have enabled ongoing prosocial, healthier behavior and support.

However, community‐level social capital (i.e., aggregate levels of social capital within adolescents' broader residential community, as determined by the “Relationships” thread from the UK Social Fabric Index), was not significantly associated with adolescent SRH in any capacity, suggesting that household‐level dimensions of social capital are more relevant for adolescent SRH than levels across the whole community. As such, there is evidence to partially support our hypothesis, that social capital would be significantly associated with higher baseline SRH and with positive changes in SRH over time. These findings align with some of the prior cross‐sectional studies of social capital (Borges et al., 2010; Novak et al., 2017; Novak et al., 2018; Winding et al., 2025), which suggests that certain dimensions of social capital (e.g. neighborhood trust, family support, civic participation) are associated with and beneficial for good adolescent SRH. Ultimately, household‐level measures of social capital played a key role in shaping and changing the course of adolescent SRH.

This distinction has significant implications for policy moving forward. Fostering local levels of social capital, such as investing in communities' social infrastructure, especially among more at‐risk groups and communities (Borkowska & Laurence, 2021; Laurence & Calvey, 2025), could protect health in times of need and facilitate healthier SRH trajectories in the long term. For adolescents specifically, it may be important for local authorities to strengthen social infrastructure, as it not only improves immediate health outcomes but also establishes foundations for their sustained well‐being throughout this developmental period. This includes investing in community‐based infrastructure (i.e., facilitating youth clubs, safe and accessible public spaces, intergenerational activities etc) to build network capital, while promoting neighborhood reciprocity and collective efficacy to enhance cognitive capital. These approaches are especially important since social capital serves as a protective buffer during crises and supports long‐term community resilience (Laurence & Calvey, 2025; Zangger, 2023; Zhang & Sung, 2023). While it must be acknowledged that there is no easy way to build social capital, as promoting social capital requires significant human and financial resources (Murayama et al., 2012), our findings imply that better local infrastructure could lead to improvements in household‐level social capital, which in turn could benefit the SRH appraisals of adolescents within the United Kingdom.

Our findings also demonstrated that, on average, SRH followed a stable linear trajectory across early and middle adolescence. The fixed effect of the slope was not statistically significant, indicating no systematic, population‐level change in SRH after adjusting for key determinants. This pattern aligns with some prior research (e.g., Breidablik et al., 2009; Fosse & Haas, 2009), while contrasting with studies that have observed non‐linear declines in mid‐adolescence (Bauldry et al., 2012; Sokol et al., 2017). The stability of SRH observed in our sample is further supported by the predominantly significant autoregressive paths, which strengthened over time. This suggests that adolescents' appraisals of their own health status became increasingly anchored to their previous assessments as they aged.

Our findings regarding the stability of SRH have pertinent implications for extant theory. The stable, autoregressive nature of SRH in our sample supports both the *enduring* and *autoregressive* components of SRH within adolescence, which previous studies have contended (Bailis et al., 2003; Boardman, 2006; Jylhä, 2009). However, the mechanism of these *enduring* or *autoregressive* components cannot be ascertained from our models. Though drawing on past research, it is likely that as adolescents aged, their SRH ratings were increasingly shaped by a cumulative internal narrative about their health, which tends to persist over time (Jylhä, 2009). This developmental process reflects the broader trajectory of self‐concept formation during adolescence, where initially fluid self‐perceptions gradually crystallize into more enduring aspects of identity (van der Cruijsen et al., 2023).

An important nuance, however, is that the variance of the slope was statistically significant, which indicates meaningful between‐individual differences in SRH trajectories. Although the average, population‐level trajectory was stable, adolescents did not follow a homogeneous pattern. Some experienced improvements in SRH, while others showed declines. Thus, while there was no evidence of systematic population‐level change, individual‐level heterogeneity suggests that SRH is not uniformly stable for all adolescents. This holds true especially when we distinguished between boys and girls and their respective SRH slopes, while girls experiencing a more severe decline in SRH than boys. This distinction underscores the importance of considering both average trends and individual variability when interpreting developmental trajectories of SRH.

Furthermore, we want to acknowledge another noteworthy finding. Higher levels of family support and life satisfaction were associated with higher baseline SRH but were also linked with small yet statistically significant declines in SRH over time. This may partially reflect regression to the mean, whereby adolescents with higher initial levels of family support and life satisfaction had greater scope for downward change over time, as scores normalized toward the population average. However, the timing of the study period may also have contributed to these patterns. The observation period overlapped with the COVID‐19 pandemic, which other studies suggest resulted in declines in adolescent mental health, particularly among those with higher pre‐pandemic well‐being and those from more advantaged households (Kleine et al., 2023; Miall et al., 2023; Reutter et al., 2024; Thygesen et al., 2021). Furthermore, our exogenous measures of household‐level social capital measures were parental/caregiver‐reported, unlike the adolescent‐reported family support and number of close friends. This may have also partially contributed to differences in findings.

Within this context, adolescents with more favorable pre‐pandemic psychosocial health may have experienced comparatively larger declines in SRH because they had further to fall, whereas those with lower baseline well‐being showed relatively minimal change. Importantly, similar effects were not observed for social capital measures: adolescents with higher social capital at baseline did not experience a decline in SRH. This finding could reflect differences in how these constructs operate. Family support and life satisfaction are proximal, subjective evaluations that are closely tied to adolescents' momentary perceptions of well‐being and may therefore be more sensitive to fluctuations during crises. In contrast, social capital, while often self‐reported, can tap into a more structural, contextual resource, which may function as a stabilizing or buffering factor during crises. Indeed, previous research suggests that social capital can mitigate the psychological impact of adverse events (Laurence, 2025; Laurence & Calvey, 2025; O'Donnell et al., 2022) and may therefore protect against declines in adolescent SRH in ways that life satisfaction and perceived family support might not. Future research could examine whether our results hold in non‐pandemic periods to assess the extent to which the effects we observe are specific to the pandemic context.

From a methodological perspective, our study also flags the importance of employing longitudinal statistical models that are nuanced enough to capture both the stable and changeable qualities of SRH. The LV‐ALT framework, which outperforms traditional growth curve and cross panel models, can specify autoregressive pathways into model estimations (Bollen & Gutin, 2021), which enables a more accurate testing of the influence that predictor variables (such as social capital) have on the outcome. However, this increased modeling flexibility is accompanied by challenges relating to model selection and the potential for overfitting. Although some prior studies reported non‐linear trajectories of SRH from mid‐adolescence into early adulthood (Bauldry et al., 2012; Sokol et al., 2017), we found that an unconditioned linear LV‐ALT model provided marginally better fit and a more parsimonious model than a quadratic growth specification. In the absence of clear improvement in model fit, adopting a more complex non‐linear growth function would increase model complexity without substantive gain and potentially lead to overfitting. If the growth function of an LV‐ALT is misspecified (e.g., linear growth forced to be non‐linear or vice versa), the autoregressive component may absorb up this misspecification, yielding to misleading autoregressive parameters and overfitting the time series structure (Ou et al., 2017; Voelkle, 2008). Furthermore, while our LV‐ALT linear model still provided good fit to the data, Bollen and Gutin (2021) acknowledged that an intercept‐only LV‐ALT model might provide even better fit for SRH data. However, since an aim of this study was to determine how social capital shapes trajectories of SRH over time, we retained the slope component to enable the examination of such change in SRH.

There are other prevailing limitations to our study design. We freely estimated residual variances, and while this approach allows for the modeling of change and stability in SRH over time, it does not permit the explicit separation of true score variance from measurement error, as would be possible with multi‐item latent constructs. Additionally, the use of a single‐item SRH measure precludes formal testing of measurement invariance across time. As a result, it cannot be definitively established whether respondents appraise their health in a comparable manner over time. Consequently, observed changes in SRH may reflect, in part, shifts in interpretation or reporting rather than true changes in underlying subjective health perceptions. Nevertheless, SRH has demonstrated strong predictive validity (Schnittker & Bacak, 2014) and is widely used in longitudinal research across developmental stages, supporting its continued use despite these limitations.

Another key limitation is that our sociodemographic and psychosocial covariates were measured at baseline only and treated as time‐invariant. Several key measures were captured at irregular intervals across UKHLS waves, preventing us from constructing meaningful time‐varying covariates. In particular, social capital was measured at baseline, whereas SRH trajectories were followed over a period that included the COVID‐19 pandemic. Social capital is unlikely to have remained static over this time period and the pandemic may have substantially altered both adolescents' social environments and their access to social resources. As such, baseline measures may not fully reflect adolescents' exposure to social capital across the study period and may have confounded longitudinal associations between social capital and adolescent SRH. The inclusion of time‐varying predictors in future research could provide richer insights into the dynamic and time‐dependent relationships between SRH and changing psychosocial circumstances. In addition, they would reduce the risk of time‐invariant unobserved heterogeneity, which may bias the current findings.

In recognition of these limitations, future research could explore several directions. Firstly, various mixture modeling approaches could identify distinct subgroups within the adolescent population that experience different SRH trajectories, moving beyond population‐level averages to understand heterogeneity in SRH. It would also be helpful to determine what factors predict group membership of potential clusters arising from mixture models. Furthermore, the significant autoregressive effects we identified suggest that failing to account for lagged SRH values in longitudinal models may result in biased estimates of other predictors' effects. Future studies should consider incorporating autoregressive components into their models when evaluating trajectories of SRH.

## CONCLUSION

Ultimately, adolescent SRH exhibits both enduring stability and a meaningful capacity for change. Our results indicate that certain psychosocial determinants, in particular social capital as reported by parents and caregivers, can partially account for change in SRH in early and mid‐adolescence, even within generally stable trajectories. These findings have important implications for public health practice and policy implementation moving forward, as it may be viable for local authorities to invest in community‐based social infrastructure and foster greater familial community engagement to improve household levels of social capital. This holds potential to not only improve adolescents' immediate health perceptions but also to promote better trajectories of perceived health, with benefits that may extend into adulthood.

## AUTHOR CONTRIBUTIONS

**Bill Calvey:** Conceptualization; methodology; investigation; validation; formal analysis; visualization; writing – original draft; writing – review and editing. **James Laurence:** Conceptualization; methodology; investigation; validation; funding acquisition; writing – original draft; writing – review and editing; formal analysis; visualization.

## FUNDING INFORMATION

This work was supported by the Economic and Social Research Council: [Grant Number ES/W00349X/1].

## CONFLICT OF INTEREST STATEMENT

The authors have no competing interests to declare.

## ETHICS STATEMENT

Ethical approval for the UK Household Longitudinal Study was granted by the University of Essex's Ethics Committee, and permission was obtained for data archival via the UK Data Service. As the present study involved secondary data analysis of anonymized publicly available data, no additional ethical approval was required.

## PATIENT CONSENT STATEMENT

Parents/guardian consent was obtained within the UK Household Longitudinal Study, with consent materials made available at: https://www.understandingsociety.ac.uk/documentation/mainstage/fieldwork‐documents.

## Supporting information


**Supplementary Materials 1.** A schematic diagram showing the screening criteria for our final sample of UKHLS adolescents.
**Supplementary Materials 2**. A list of all measures included in our analyses (i.e., exposures, covariates, and outcome variables).
**Supplementary Materials 3**. Linear Latent Variable Autoregressive Trajectory models with interaction terms between gender and different dimensions of household and community‐level social capital, all tested individually. All covariates were included but not shown in the table below.
**Supplementary Materials 4**. A Linear Latent Variable Autoregressive Trajectory model, with a combined household‐level social capital score, instead of two separate “cognitive” and “network” social capital indices. All covariates were included but not shown in the table below.
**Supplementary Materials 5**. Baseline descriptive statistics for the full sample (*n* = 2413).

## Data Availability

The data that support the findings are available upon registration with the UK Data Service, subject to standard checks: https://ukdataservice.ac.uk/. Code that supports the findings of this study is available from the corresponding author upon reasonable request.
