# Supplementary material for: How does social capital shape trajectories of adolescent self‐rated health in the United Kingdom?
Source: J Res Adolesc. 2026 Jul 21;36(3):e70238. doi: 10.1111/jora.70238 (PMC13388828; doi:10.1111/jora.70238)
Supplement: Supplementary file 1 — Supplementary Materials 1. A schematic diagram showing the screening criteria for our final sample of UKHLS adolescents. Supplementary Materials 2. A list of all measures included in our analyses (i.e., exposures, covariates, and outcome variables). Supplementary Materials 3. Linear Latent Variable Autoregressive Trajectory models with interaction terms between gender and different dimensions of household and community‐level social capital, all tested individually. All covariates were included but not shown in the table below. Supplementary Materials 4. A Linear Latent Variable Autoregressive Trajectory model, with a combined household‐level social capital score, instead of two separate “cognitive” and “network” social capital indices. All covariates were included but not shown in the table below. Supplementary Materials 5. Baseline descriptive statistics for the full sample (n = 2413). [file JORA-36-0-s001.docx]

**Supplementary Materials 1**

A schematic diagram showing the screening criteria for our final sample of UKHLS adolescents.


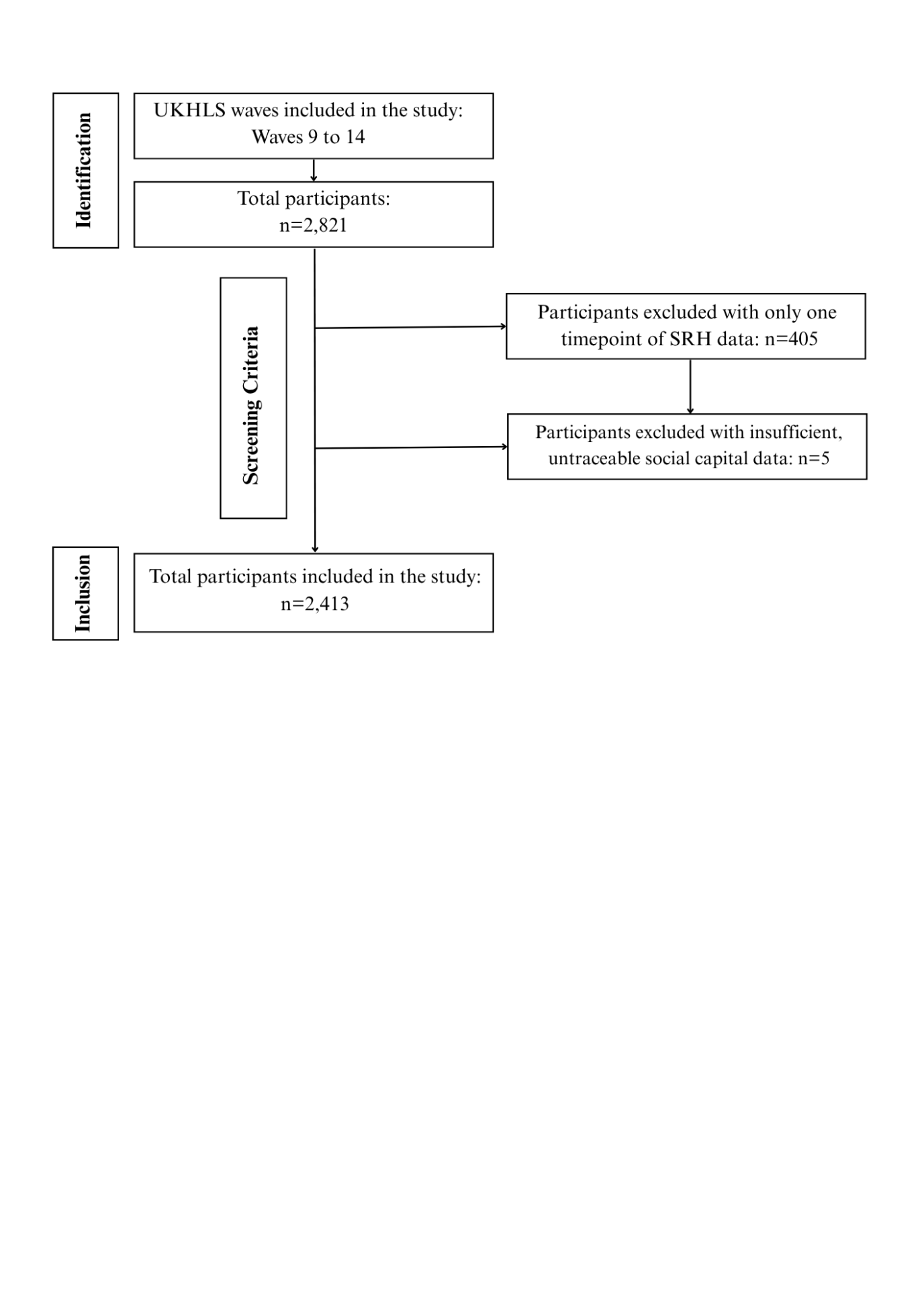


**Supplementary Materials 2**

A list of all measures included in our analyses (i.e., exposures, covariates and outcome).

| **Measure** | **Type** | **Label** | **Value** |
| --- | --- | --- | --- |
| Age | Covariate |  | 0 to 21 |
| Sex | Covariate |  | Male; female. |
| Ethnicity | Covariate |  | White; Black; Asian; Mixed; Other. |
| Economic disadvantage | Covariate |  | -2 to 2.5 |
| Parental education | Covariate | What is the highest level of education you obtained? | None/Primary; Lower secondary; Upper secondary; Tertiary |
| Change in address | Covariate |  | No; Yes. |
| Single parent household | Covariate |  | Not a single parent household; single parent household. |
| Limiting health condition/disability | Covariate | Long-term health problem or disability | No; limited a little; limited a lot |
| Family support | Covariate | Do you feel supported by your family, that is the people who live with you? | I feel supported by my family in **most or all** of the things I do (=1); I feel supported by my family in **some of the things** I do (=2); I **do not** feel supported by my family in the things I do (=3) |
| Life satisfaction | Covariate | How do you feel about your life as a whole? | 1 (completely happy) to 7 (not happy at all) |
| Close friends | Covariate | How many close friends do you have? | 0 to 10 |
| Household-level social capital |  |  |  |
| *Cognitive* | Exposure | - I feel like I belong to this neighbourhood. - I plan to remain a resident of this neighbourhood for a number of years. - I think of myself as similar to the people that live in this neighbourhood. | Strongly agree (=1); agree (=2); neither agree nor disagree (=3); disagree (=4); strongly disagree (=5) |
| *Network* | Exposure | - The friendships and associations I have with other people in my neighbourhood mean a lot to me. - If I needed advice about something I could go to someone in my neighbourhood. - I borrow things and exchange favours with my neighbours. - I would be willing to work together with others on something to improve my neighbourhood. - I regularly stop and talk with people in my neighbourhood. | Strongly agree (=1); agree (=2); neither agree nor disagree (=3); disagree (=4); strongly disagree (=5) |
| Community-level social capital | Exposure | UK Social Fabric Index: Relationships dimension | 1.5 to 6.5 |
| Self-rated Health | Outcome | In general, would you say your health is... | Poor (=1); fair (=2); good (=3); very good (=4); excellent (=5). |

| **Supplementary Materials 3**  Linear Latent Variable Autoregressive Trajectory models with interaction terms, between sex and different dimensions of household and community-level social capital, all tested individually. All covariates were included but not shown in the table below. | | |
| --- | --- | --- |
|  | *Estimate* | *95% CI* |
| **Intercept** |  |  |
| Sex*Network Social Capital | -.02 | (-.09 / .07) |
| **Slope** |  |  |
| Sex*Network Social Capital | -.01 | (-.03 / .01) |
| **Intercept** |  |  |
| Sex*Cognitive Social Capital | -.03 | (-.12 / .06) |
| **Slope** |  |  |
| Sex*Cognitive Social Capital | -.02 | (-.04 / .01) |
| **Intercept** |  |  |
| Sex*Community-level Social Capital | .03 | (-.03 / .09) |
| **Slope** |  |  |
| Sex*Community-level Social Capital | .001 | (-.02 / .02) |

*Note:* Significant at ∗p< .05, ∗∗p< .01, ∗∗∗ p< .001.

| **Supplementary Materials 4**  A Linear Latent Variable Autoregressive Trajectory model, with a combined household-level social capital score, instead of two separate ‘cognitive’ and ‘network’ social capital indices. All covariates were included but not shown in the table below. | | | |
| --- | --- | --- | --- |
|  | *Estimate* | *Standardised Estimate* | *95% CI* |
| **Intercept** | | | |
| Overall Household-level Social Capital | 0.05** | .06 | (.01 / .09) |
| **Slope** |  |  |  |
| Overall Household-level Social Capital | .01 | .05 | (-.01 / .02) |
| **Intercept** |  |  |  |
| Community-level Social Capital | -.01 | .02 | (-.06 / .03) |
| **Slope** |  |  |  |
| Community-level Social Capital | -.01 | .01 | (-.02 / .01) |

*Note:* Significant at ∗p< .05, ∗∗p< .01, ∗∗∗ p< .001.

| **Supplementary Materials 5**  Baseline descriptive statistics for the full sample (n=2413). | | | |
| --- | --- | --- | --- |
| **Variable** | **Mean (SD) / %** | **N (non-missing)** | **% (missing)** |
| **Age** | 12.49 (1.70) | 0 | 0% |
| **Sex** |  | 0 | 0% |
| Male | 48.74% |  |  |
| Female | 51.26% |  |  |
| **Parental Education** |  | 2175 | 9.6% |
| None | 14.53% |  |  |
| Lower Secondary | 22.99% |  |  |
| Upper Secondary | 8.87% |  |  |
| Tertiary | 53.61% |  |  |
| **Ethnicity** |  | 3 | 0.12% |
| White | 72.65% |  |  |
| Black | 4.68% |  |  |
| Asian | 16.37% |  |  |
| Mixed | 5.56% |  |  |
| Other | 0.62% |  |  |
| **Self-rated Health** | 3.93 (0.92) | 0 | 0% |
| **Single Parent Household** |  | 2353 | 2.25% |
| Yes | 17.55% |  |  |
| No | 82.45% |  |  |
| **Change of Address** |  | 0 | 0% |
| Yes | 5.93% |  |  |
| No | 94.07% |  |  |
| **Economic Disadvantage Score** | 0.07 (0.97) | 2313 | 0.04% |
| **Limiting Health Condition/Disability** |  | 2387 | 1.08% |
| None | 88.81% |  |  |
| Yes, limited a little | 9.26% |  |  |
| Yes, limited a lot | 1.93% |  |  |
| **Family Support** | 2.81 (0.42) | 2388 | 1.04% |
| **Life Satisfaction** | 5.76 (1.2) | 2392 | 0.87% |
| **Close Friends** | 5.48 (2.96) | 2365 | 1.99% |
| **Household-level Cognitive Social Capital** | -0.01 (0.74) | 2368 | 1.86% |
| **Household-level Network Social Capital** | 0.03 (0.8) | 2367 | 1.91% |
| **Community-level Social Capital** | 3.6 (0.85) | 2313 | 0.04% |
